# A new bin size index method for statistical analysis of multimodal datasets from materials characterization

**DOI:** 10.1038/s41598-023-37969-2

**Published:** 2023-07-05

**Authors:** Tao Jiang, Shengmin Luo, Dongfang Wang, Yucheng Li, Yongkang Wu, Li He, Guoping Zhang

**Affiliations:** 1 Department of Civil and Environmental Engineering, University of Massachusetts Amherst, Amherst, MA 01003 USA; 2 Department of Civil and Environmental Engineering, University of Wisconsin-Madison, Madison, WI 53715 USA; 3 Key Laboratory of Geotechnical and Underground Engineering of Ministry of Education, Tongji University, Shanghai, 200092 China; 4 School of Civil Engineering, Beijing Jiaotong University, Beijing, 100044 China

**Keywords:** Civil engineering, Theory and computation

## Abstract

This paper presents a normalized standard error-based statistical data binning method, termed “bin size index” (BSI), which yields an optimized, objective bin size for constructing a rational histogram to facilitate subsequent deconvolution of multimodal datasets from materials characterization and hence the determination of the underlying probability density functions. Totally ten datasets, including four normally-distributed synthetic ones, three normally-distributed ones on the elasticity of rocks obtained by statistical nanoindentation, and three lognormally-distributed ones on the particle size distributions of flocculated clay suspensions, were used to illustrate the BSI’s concepts and algorithms. While results from the synthetic datasets prove the method’s accuracy and effectiveness, analyses of other real datasets from materials characterization and measurement further demonstrate its rationale, performance, and applicability to practical problems. The BSI method also enables determination of the number of modes via the comparative evaluation of the errors returned from different trial bin sizes. The accuracy and performance of the BSI method are further compared with other widely used binning methods, and the former yields the highest BSI and smallest normalized standard errors. This new method particularly penalizes the overfitting that tends to yield too many pseudo-modes via normalizing the errors by the number of modes hidden in the datasets, and also eliminates the difficulty in specifying criteria for acceptable values of the fitting errors. The advantages and disadvantages of the new method are also discussed.

## Introduction

Variates or random variables obeying multimodal distributions are ubiquitous and frequently encountered in all science and engineering disciplines^1–4^. If the mathematical distribution function is known, the graphic presentation of such a distribution, such as the plot of probability density function (PDF)^5,6^, is typically characterized by multiple isolated or overlapping distinct peaks or modes. In most cases, however, the actual mathematical distribution function is not readily available^7^ for an unknown variate, and hence multiple, repeated sampling or measurements are necessary to obtain a series of data, which vary with the outcomes of random events or phenomena and hence constitute a multimodal random dataset^7–9^. There are numerous examples of multimodal datasets encountered in daily practices and routine measurements. For instance, the particle size distribution (PSD) of a dry concrete mix that is usually composed of finer Portland cement, medium-sized sand, and coarser aggregate as well as additives such as plasticizers exhibits at least three modes. Moreover, upon hydration and setting, the hardened concrete made of inter-cemented particles may still possess a multimodal (e.g., 3 or more modes) PSD, due to the formation of nanocrystalline calcium-silicate-hydrates, non-hydrated cement particles, and non-reactive sands and aggregates. The second example is the characterization spectra, obtained by Fourier transform infrared (FTIR) and solid-state nuclear magnetic resonance (ssNMR) spectroscopy, of some silicates (e.g., silica aerogels)^10^ and aluminosilicates (e.g., geopolymers)^11,12^ consisting of highly complex networks of molecular clusters including tetrahedral [SiO_4_]^4−^ and/or [NaAlO_4_]^4−^. Other examples include the PSD and pore size distributions of some natural soils and suspended sediments^13,14^ consisting of different-sized particle groups (e.g., clay, silt, sand, and clay flocs), as well as the small (e.g., nano or micro) scale local mechanical properties of multiscale and multiphase composite materials consisting of mechanically distinct constituents (e.g., rocks, concretes), as further discussed later.

A thorough understanding of a multimodal dataset repeatedly sampled from a variate requires further data processing and treatment to uncover the basic statistics and underlying distributions^4,15,16^. Such data analyses usually commence with assuming a certain type of statistical distributions (e.g., the Gaussian or normal distribution) for the variate of interest and terminate with statistical deconvolution, which is widely used to definitely determine the number, mean, standard deviation (SD), and fraction of different modes as well as the global distribution function.

Depending upon the nature of the datasets (e.g., one, two, or multi-dimensional datasets), PDF, cumulative distribution function (CDF), and Gaussian mixture modeling (GMM)-based statistical deconvolutions are well-developed and readily available at present. For example, numerous work involving one or more of the aforementioned deconvolution methods has been reported for characterizing the mechanical properties of multiphase composites using nano/micro indentation^12,17–21^. In fact, most of the aforementioned materials characterization problems usually involve only univariate datasets, offering the PDF-based deconvolution certain advantages over the CDF and GMM. For example, PDF is more visually intuitive than the CDF in terms of the deconvoluted distinct modes, since the mean (i.e., center) and SD (i.e., width) can be directly identified from the plot, while the area under each deconvoluted peak represents its global makeup percentage relative to other modes. In fact, this is probably the same reason why the PDF is the most widely used or preferred mathematical function of a variate, and hence the PDF plays a vital role in data processing and analyses. The PDF is typically used to account for the likelihood or probability of the variate taking a specific value that is within a particular range, but not one particular value. Such a probability is defined by the area under the PDF confined between the data range.

The PDF-based deconvolution starts first with the construction of a histogram, or a discretized graphical presentation of the dataset of more or less continuous numbers, which is the most widely used and straightforward graphical description of a dataset. Histogram construction in turn requires data binning, a pre-processing technique for grouping the datasets into a smaller number of bins. In other words, a properly selected, rational bin size/width (*b*) is necessary to bin and group the individual measurement data into different intervals so that the number of data falling within each interval (i.e., bin) can be counted and hence the occurrence frequency within each bin determined. It is only after an appropriate and rational bin size or number of bins (*N*_b_) is determined that a truly representative histogram can be obtained. In general, the bins must be adjacent with an equal size, but sometimes unequal bin sizes are necessary for processing the statistical data of a variate^22–24^. Different bin sizes can reveal different features of the measurement data. Wider bins can accommodate more data, leading to a smaller number of bins or the underlying occurrence frequency bars, and hence reduce the noise due to the sampling randomness, a phenomenon also called “oversmoothing”^25–27^. In contrast, narrow bins result in more underlying occurrence frequency bars, and hence the histogram is more sensitive to the sampling noise, or “undersmoothing”^26,28,29^. Therefore, varying the bin size for a given dataset can result in different histograms and differently fitted, or sometimes misleading, PDFs with varying degrees of error.

However, to date, the method to determine the “best” number of bins or a rational, optimal bin size is not yet universally available or accepted^4,16,30^. Many attempts have been made in the past by theoreticians or statisticians to propose different criteria to estimate the optimal bin sizes^31–36^ required for data binning and subsequent histogram construction or PDF deconvolution. Most of these binning methods suggest a bin size that is highly dependent upon the total number (*n*), and to a lesser extent the maximum and minimum, of the measurement data in the dataset. In particular, the Freedman-Diaconis rule^31^ yields a relatively better estimate of the bin size due to the adoption of the interquartile range (*IQR*), which makes the estimated *b* less sensitive to the maximum and minimum but the standard deviation of the dataset. Sturges’ rule derived from a binomial distribution assumes an approximately normal distribution and implies that the bin size depend on the range of the data^37^. Scott’s rule also is optimal for random variables of normally distributed data^30^. More recently, the Shimazaki–Shinomoto rule considers a rick function that is based on the minimal mean integrated squared error (MISE), a measure of the goodness of the fit of a trial histogram to the unknow PDF function, to estimate the optimal bin size^38–40^. Of these proposed rules, some may rely on the assumption of a normal distribution and hence cannot be generalized to other data with multiple modes, while others might overfit the histogram with too many modes that tend to yield the smallest fitting error. In summary, despite of these proposed rules, selection of a rational bin size is still largely empirical and mostly a matter of individual choice, but not objective or free of personal judgment and experience.

In this paper, a new “data binning” approach, termed the “Bin Size Index (BSI)” method, is proposed to determine the optimal bin size required for the construction of histograms of multimodal datasets that are then used for PDF-based deconvolution. A wide variety of datasets were examined, including four normally-distributed synthetic datasets with varying the number, mean, SD, and fraction of different modes, three normally-distributed, real measurement datasets on the small-scale, local Young’s moduli of three sedimentary rocks obtained by nanoindentation, and three log-normally distributed datasets on the PSD of flocculated illite suspensions. Validation of the fitting accuracy by the synthetic datasets with normal and lognormal distributions can expectedly manifest the effectiveness of the BSI method in selecting an optimal bin size for constructing the histograms to be used subsequently for the PDF-based statistical deconvolution, especially for multiphase materials testing. This process also leads to the clearly and separately identified individual modes as well as the determination of the number, mean, SD, and fraction of each identified mode contained in the statistical dataset, of which the mean and SD are valid only for statistical distributions with symmetrical PDFs. This new method particularly penalizes the overfitting that tends to yield too many pseudo-modes via normalizing the errors by the number of modes hidden in the datasets, and also eliminates the difficulty in specifying criteria for acceptable values of the fitting errors.

## The new binning method

The generalized problem of data binning is first described here, followed by the detailed concepts and algorithms of the new BSI method. To date, a broad and diverse spectrum of statistical functions have been discovered or formulated to mathematically describe the randomness of natural processes, phenomena, and observations, most of which tend to obey heavy-tailed distributions^41,42^, such as social sciences laws^43^, streamflow^44^, and financial modeling^45^, among others. However, the normal or Gaussian distribution plays an essential role in approximating other fundamental statistical distributions (e.g., binomial distribution, Poisson distribution, chi-squared distribution, Student’s *t*-distribution). Moreover, it is one of the simplest and widely used statistical functions accounting for various natural and artificial phenomena (e.g., the height patterns of specific populations can be mostly modeled with a normal distribution, the size of living tissues basically follows a lognormal distribution but can be described by a normal distribution after the logarithmic transformation), particularly for the measurements of materials properties (e.g., strength, elastic modulus). A multimodal variate *x* can be assumed to obey a Gaussian distribution, and its *K*-mode (where *K* is the number of mode) PDF can be written as:

1 $$f\left( x \right) = \sum\limits_{j = 1}^{K} {A_{j} f_{j} \left( {x|\mu_{j} ,\sigma_{j} } \right)}$$

where *f*(*x*) and *f*_j_(*x*) (*j* = 1, 2, 3, …, *K*) are the global and individual mode’s Gaussian PDFs respectively; *A*_j_, *μ*_j_, and *σ*_j_ the fraction, mean, and SD of the *j*-th mode, respectively. Overall, the coefficients *A*_j_ are constrained by:

2 $$\sum\limits_{j = 1}^{K} {A_{j} = 1.0}$$

Appropriate sampling or measurement of the variate *x* repeated by *n* times results in a multimodal dataset ***X***, a one-dimensional matrix with *n* elements. To better understand the variate *x*, the dataset ***X*** needs to be analyzed statistically, particularly for the determination of the statistical properties of all modes, including *K*, *A*_j_, *μ*_j_, and *σ*_j_, via statistical deconvolution. As stated earlier, PDF-based deconvolution is typically preferred over other counterparts, which starts with the selection of an appropriate bin size *b* to construct the histogram of the dataset ***X***, *H*(***X***). The goal of deconvolution is to minimize the error *E*(*x*) between the real but unknown analytical PDF *f*(*x*) and the histogram *H*(***X***):

3 $$E\left( x \right) = f\left( x \right) - H\left( {\rm X} \right) = E\left( {b,K,A_{j} ,\mu_{j} ,\sigma_{j} } \right)$$

That is, the error *E*(*x*) is a function of five variables shown in the above equation, of which the *b* is the most important since it is necessary to initiate the deconvolution.

The newly proposed BSI method consists of a series of computational algorithms leading to the selection of the optimal bin size, *b*_opt_. First, the Freedman-Diaconis rule^31^ is adopted to generate an initial trial bin size *b*_0_ for the dataset ***X***:

4 $$b_{0} = \frac{{2 \times IQR\left( {\rm X} \right)}}{{n^{\frac{1}{3}} }}$$

where *IQR* is the interquartile range of the considered dataset. Then six or more different trial bin sizes, *b*_m_ (*m* = 1, 2, 3, …, 6) < *b*_0_, are selected, preferably covering a wide range with a small but equal difference:

5 $$\Delta b = b_{m + 1} - b_{m}$$

The reason for *b*_m_ < *b*_0_ is that larger bin sizes always result in a relatively higher normalized standard error and smaller BSI (as defined later), according to preliminary work. Therefore, the *b*_0_ determined by the Freedman–Diaconis rule^31^ can serve as a starting upper bound for other trial bin sizes *b*_m_.

Each of the seven different bin sizes is then used to construct its own histogram, followed by statistical deconvolution with widely and commercially available software, such as PeakFit^20,46^. In this study, however, fitting the histogram with PDF was performed by the OriginPro (Version 2020 (9.7)) Peak Analyzer (OriginLab Corporation, USA) that can perform peak fitting under the assumption that the variate *x* obeys a multimodal Gaussian distribution described by Eq. (1).

Particularly noteworthy is that the variable *K*, the total number of modes, is not independent, but varies with the bin size *b*. For a given histogram, the mode *K* should in theory be varied to find the best fit PDF by minimizing the *E*(*x*), starting from *K* = 1 to a maximum *K*_max_. In many cases, however, the modes of a histogram constructed for a given *b* may be visually identified and discerned, or are clearly separated apart, and hence there is no need to vary *K* further in the deconvolution. The *K*_max_ is determined by two conditions (as discussed later): (1) it is constrained by the degree of freedom, and (2) for real measurement data, other accompanying results (e.g., the mineralogical composition of a rock determined by X-ray diffraction) may also be used to aid the selection of *K*_max_.

This step of histogram fitting yields a certain number of modes *K*, as well as the mean, SD, and fraction of each mode. As such, the overall analytical (or deconvoluted) multimodal PDF (i.e., Eq. (1)) encompassing *K* modes can be determined. Usually this PDF does not match exactly with the histogram constructed by the particular bin size *b.* To quantify the goodness of fit or the error between the analytical PDF and experimental histogram, the least-squares criterion, a routine method used in most statistical analysis for minimizing the sum of squares due to error (*SSE*), is herein performed:

6 $$SSE = \sum\limits_{i = 1}^{{N_{b} }} {w_{i} \left( {\hat{y}_{i} - y_{i} } \right)^{2} }$$

where *N*_b_ is the total number of bins, *y*_i_ and *ŷ*_i_ are the *i-*th bin occurrence frequency or probability of the experimental histogram and analytical PDF respectively, and *w*_i_ is the weight applied to the *i*th bin, which is usually equal to 1.0. Since defining a *K*-mode normal distribution function requires 3 *K* parameters (i.e., the variables *A*_j_, *μ*_j_, and *σ*_j_ shown in Eq. (1), where *j* = 1, 2, 3, …, *K*) and the sum of all fractions, *A*_j_, should equal to 1.0 (i.e., Eq. (2)), the latter of which also serves as an additional constraint, the degree of freedom (*DOF*) is defined:

7 $$DOF = N_{b} - 3K - 1$$

The standard error (*S*_E_) for each trial bin size can then be calculated by:

8 $$S_{E} = \sqrt {\frac{SSE}{{DOF}}}$$

To obtain a rational *S*_E_, the *DOF* must be an integer greater than or equal to 1, or (*N*_b_ – 3 *K* − 1) ≥ 1. As such, *K* ≤ (*N*_b_ – 2)/3 or the maximum *K* should be *K*_max_ = (*N*_b_ – 2)/3. The above process is repeated for all trial bin sizes, resulting in multiple *S*_E_ values with a mean *μ*_S_ and an SD *σ*_S_. In consideration of the potential variations in the *S*_E_ values obtained from all different trial bin sizes, which can differ by more than one order of magnitude and are much smaller than 1.0, a step of mean normalization of *S*_E_ is further proposed to obtain the mean-normalized residual standard error *S*_EN_ whose values are scaled up to ~ 1.0:

9 $$S_{EN} = \frac{{\left| {S_{E} - \mu_{S} } \right|}}{{\sigma_{S} }}$$

Finally, a new quantitative parameter, termed as “bin size index” (*BSI*), is defined for the first time in this paper to further evaluate the accuracy of the above deconvolution and curve fittings for each of the trial bin sizes:

10 $$BSI = \frac{{\left| {2 \times {\text{ln}}\left( {{\text{S}}_{{{\text{EN}}}} } \right)} \right|}}{K}$$

In general, *S*_EN_ is much less than 1.0, and hence ln(*S*_EN_) is always negative, which is why the absolute value function is used to define the BSI. As such, the smaller the *S*_EN_, the greater the BSI. Clearly, the determined BSI values based on the above equations are primarily regulated by the *S*_E_ obtained at each bin size. Therefore, a finite number of modes *K* ≤ *K*_max_ can be tried at each trial bin size and the one yielding the highest BSI is selected as the correct number of modes deconvoluted at this particular trial bin size. Then plotting the BSI against *b* yields a unimodal peak or multiple peaks, and the peak with the highest BSI should be used to find the *b*_opt_. An example is given later (“Analyses of datasets on rocks’ elasticity”) for the dataset obtained from nanoindentation testing of a shale sample to determine its multiphase elastic properties.

In summary, each *S*_EN_ is objectively determined by comparing individual *S*_E_ and the mean *μ*_S_ of all *S*_E_ values, followed by the normalization by the standard deviation *σ*_S_. As discussed in the analysis of real measurement data, quite often, the lowest *S*_EN_ usually corresponds to the highest BSI, which corresponds to the *b*_opt_. In other words, the *BSI* is intentionally penalized by the number of modes *K* so that the normalized standard error is shared by the number of modes *K*. All other statistical parameters, including *K*, *A*_j_, *μ*_j_, and *σ*_j_ in Eq. (1) can be determined by deconvoluting the histogram constructed by the *b*_opt_. Although other trial bin sizes are used to construct different histograms that are also deconvoluted, their results are used to calculate the *BSI* and hence aid the selection of the maximum *BSI* and hence the *b*_opt_. In fact, the underlying concept of *BSI* is inspired by the maximum likelihood-based criteria such as the Akaike information criterion (AIC)^47^ and Bayesian information criterion (BIC)^48^.

## Data description and collection

### Synthetic datasets

The general statistical accuracy and effectiveness of the proposed BSI method were first investigated and validated by synthetic datasets that were generated by a random number generator (i.e., the built-in “Random Number Generation” data analysis tool) in the Microsoft Excel program (Microsoft Corporation, USA). First, several individual unimodal subdatasets, each of which corresponded to one particular mode of a variate obeying a unimodal normal distribution, were generated under the preset statistical parameters, including the number of data entries, mean, and SD of the normal distribution. Then a selected number (i.e., the mode number *K* = 3 or 5) of different subdatasets were merged into one integrated master dataset, which became a multimodal distribution. In total, four different multimodal datasets (i.e., identified by I, II, III, or IV) with varying the number, mean, SD, and fraction of all modes were created for different purposes (Table 1). For example, while Datasets I and II consist of 3 modes, the rest two have 5 modes. On the other hand, all modes of Datasets I and III had equal fractions (i.e., each mode has 1,000 and 3,000 data entries for Datasets I and III respectively), the other two counterparts have unequal fractions of different subdatasets (Table 1).

**Table 1:** Summary and comparison of the pre-set versus deconvoluted statistical parameters for the four synthetic multimodal datasets following a normal distribution.

Dataset ID (type)	Mode ID (# of data)	Mean (*μ*)		Standard deviation (*σ*)		Fraction (*A*) (vol.%)	
		Pre-set	Deconvoluted	Pre-set	Deconvoluted	Pre-set	Deconvoluted
I (equal fraction)	1 (1000)	200.00	202.30	10.00	10.29	33.33	33.81
	2 (1000)	220.00	233.18	10.00	9.79	33.33	38.10
	3 (1000)	240.00	255.60	15.00	13.66	33.33	28.09
II (unequal fraction)	1 (3000)	300.00	300.00	10.00	10.23	50.00	50.00
	2 (1000)	320.00	320.00	15.00	12.81	16.67	16.70
	3 (2000)	340.00	340.00	30.00	28.85	33.33	33.30
III (equal fraction)	1 (3000)	200.00	203.83	10.00	10.32	20.00	20.05
	2 (3000)	300.00	303.09	20.00	19.77	20.00	19.43
	3 (3000)	360.00	364.21	18.00	19.65	20.00	21.17
	4 (3000)	400.00	404.04	15.00	15.26	20.00	19.26
	5 (3000)	450.00	451.15	21.00	21.49	20.00	20.08
IV (unequal fraction)	1 (3000)	200.00	201.88	10.00	10.30	15.00	15.20
	2 (2000)	250.00	251.02	20.00	18.52	10.00	9.48
	3 (4000)	310.00	312.59	15.00	15.81	20.00	21.86
	4 (5000)	360.00	363.40	25.00	22.95	25.00	22.93
	5 (6000)	415.00	416.65	18.00	17.79	30.00	30.53

To guarantee sufficiently distinguished contrast between any two adjacent modes (i.e., by preventing excessive overlap of two neighboring unimodal distributions so that the two modes would not be treated as one single mode)^49^ , a constraint was added to the data generation:

11 $$\mu_{j} + \sigma_{j} < \mu_{j + 1} + \sigma_{j + 1} ,\quad j = { 1},{ 2}, \, \ldots ,K$$

The proposed BSI method was then applied to process and deconvolute the four multimodal datasets so that the statistical parameters, including the number of modes, mean, SD, and fraction of each mode, were determined. During the deconvolution, although the number of modes *K* was known, the range of tried *K* values was intentionally increased, i.e., *K* = 1 to 6 (= *K*_max_) for Datasets I and II and 1 to 7 (= *K*_max_) for Datasets III and IV, to examine the BSI method’s effectiveness in determining the correct number of modes. In addition, many different initial values of the mean, SD, and fraction of all modes were needed to start with the fitting, and a proper histogram could aid the selection of these initial values.

### Datasets on the elasticity of rocks

As the first example, the datasets on the elastic modulus of natural rocks were used to validate the viable applications of the BSI method. Heterogeneous composite materials usually consisting of multiple, compositionally distinct but structurally-integrated constituent phases across different scales (i.e., from nano- to macro-scales) are extensively manufactured and widely used for various functionalities in practice, such as metal foams^50^, ceramics^51^, polymers and biomaterials^52^, and Portland cement-based concretes. Besides the artificially engineered composites, naturally occurring counterparts are also abundant and frequently encountered in human activities, such as rocks and woods. One feature common to all these materials is that their mechanical properties (e.g., strength, stiffness) are affected by the properties of individual constituents and the fashion through which these constituents interact across different scales (i.e., from the nanoscale particles at the order of ~ tens of nanometers to a few micrometers, to mesoscale structural units, and to macroscale bulk material at the length scale of a few millimeters to meters). For example, as a representative kind of fine-grained sedimentary rocks, shales are formed by the deposition of primarily cohesive suspended sediments (e.g., clay minerals) together with minor coarse-grained sand and silt particles in natural water environments (e.g., rivers, oceans), followed by subsequent long-term (e.g., millions of years) geological processes (e.g., compaction, consolidation, diagenesis, tectonic stressing), during which a complex composite structure is developed involving multiple mineral constituents and associated interactions such as degree of packing, particle arrangements, interparticle contacts, and cementation^21,53–55^. As such, shales are typically made of a fine-grained, relatively homogeneous, clay mineral-based matrix and other hard minerals as solid inclusions randomly distributed within the matrix. Characterizing the in-situ mechanical properties of the matrix and the solid inclusions requires small-scale mechanical testing techniques such as nanoindentation.

Since its initial inception^56^, statistical grid nanoindentation has evolved as a convenient, fast experimental technique to probe the in-situ mechanical properties such as elasticity and hardness of individual constituent phases of multiphase composites such as rocks and concretes. More recently, big data-based nanoindentation has also been developed to characterize the cross-scale elasticity of shales and sandstones, i.e., the elastic moduli of individual constituents at the nano/micro scale and of bulk rock as a composite at the meso/macro scale^20^. One key hypothesis for this technique is that each of the probed mechanical properties of individual phases is a multimodal variate obeying a normal distribution, and thus a massive number of measurements required to ensure statistical accuracy, particularly for the determination of the fraction of different constituent phases, constitute a multimodal dataset. For instance, the Young’s modulus data obtained by nanoindentation randomly probing numerous locations on the sample surface constitute a multimodal dataset. The utmost important step for this grid nanoindentation approach is the statistical deconvolution of the dataset to determine the number, mean, SD, and fraction (i.e., in terms of area or volume) of all different modes or constituent phases of a composite. To date, various statistical analysis methods have been developed, including the K-means clustering, PDF, CDF and multivariate GMM, of which the PDF-based deconvolution is the most widely used, most likely due to its easy implementation, straightforward concepts, and intuitive graphic presentation. However, the PDF-based deconvolution is conducted on the experimental histograms whose construction requires the selection of an appropriate and rational bin size (or equivalently the number of bins).

To validate the effectiveness of the BSI methods in characterizing the mechanical properties of multiscale, multiphase sedimentary rocks, grid nanoindentation measurements on two sandstones (hereafter abbreviated as KS-45 and KS-52) and one shale (hereafter referred to as Longmaxi shale due to its origin) were selected and analyzed. Experimental details on the samples, sample preparation methods, and measurement procedures can be found in prior publications:^46^ for the two sandstones, and^19^ for the shale. In brief, nanoindentation testing was conducted on the highly polished disk specimens in a Keysight G200 nanoindenter (Keysight Technologies, Inc., Santa Rosa, Ca) equipped with a Berkovich diamond indenter with a tip radius of < 20 nm under the continuous stiffness measurement (CSM) mode. As such, the Young’s modulus from each of the ~ 1029 indents was continuously obtained over the entire indentation depth of up to ~ 8 μm. As examples, the datasets for the Young’s modulus extracted at the indentation depth of 500, 150, and 350 nm for the KS-45, KS-52, and Longmaxi shale, respectively, were selected for statistical analysis. Further noteworthy is that the mineralogical compositions of these three rocks were also analyzed by qualitative and quantitative X-ray powder diffraction (XRD)^19,46^. Such results can be used to partially validate the accuracy of the proposed BSI method, since the statistical deconvolution can also yield the quantitative fractions of different mineral constituents in these rocks. With the XRD results, the *K*_max_ was pre-set as 9, 7, and 8 for the KS-45, KS-52, and shale, respectively.

### Datasets on the PSD of flocculated clay suspensions

The second representative example used to illustrate the BSI method’s applicability is the PSD of flocculated clay suspensions. Natural waters are usually loaded with suspended cohesive sediments, which are, unlike the sand/silt or other coarse-grained sediments, primarily composed of different types of platy-shaped clay minerals with nano and submicrometer sizes (e.g., < 2 μm), large specific surface areas, and chemically active surfaces (i.e., permanent negative charges on face surface and pH-dependent charges on edge surface)^57,58^. These features lead to intensive interactions (e.g., flocculation and aggregation) among primary clay particles themselves and other suspended particulate matter (e.g., extracellular polymeric substances, EPS) that also has surface charges or chemically active functional groups, resulting in complex, multimodal PSD with different-sized particle groups. For instance, prior work showed that primary clay particles, flocculi, microflocs, and macroflocs could form as a result of counterbalanced effects of continuous flocculation versus breakage in the hydrodynamic water environments^13,14,59^. Knowledge of the complex PSD of suspended sediments is of essential importance for sediment and coastal shoreline management, and predictive modeling of sediment transport and fate, which play a vital role in the sustainable development and management of natural water environments^60^. Moreover, it is well known that the PSD of naturally occurring soils, deposits, and suspended sediments can be best described by a lognormal distribution. To further validate the applicability and performance of the BSI method for binning lognormally distributed, multimodal datasets, experiments were designed and conducted to obtain the PSD data of flocculated clay suspensions.

High-purity illite (IMt-1) acquired from the U.S. Clay Minerals Society Source Clay Repository (Purdue University, Indiana, USA) was used to prepare flocculated clay suspensions for PSD measurements, owing to its abundance in terrestrial and marine cohesive sediments. The as-received rock chips of illite were first wet-ground to a fine powder of < 20 μm in size. Following previously developed procedures^61^, the ground illite of 0.12 g was first soaked in a centrifuge tube filled with 10 mL deionized (DI) water for > 16 h, followed by mixing for 10 min in a blender with additional 290 mL DI water, resulting in an illite suspension of 0.4 g/L in concentration. The purpose of these disintegration and dispersion steps was to obtain the truly primary clay particles for the formation of representative illite flocs, instead of the pre-existing aggregates in the samples.

To promote flocculation and simulate the hydrodynamic shearing in turbulent flows, the above clay suspensions with an original pH 8.61 were altered to have a 35 ppt NaCl salinity and three pH values, 8.61, 4.51, 2.25, the latter of which was adjusted through titration by 0.1 M HCl solution, since clay minerals such as illite tend to form repulsion in a clean or basic suspension. Flocculation of the suspended illite particles was then achieved by continuously vibrating at a speed of 150 oscillations/min for 24 h the aforementioned clay suspension in a Burrell Model 95 wrist-action shaker (Burrell Scientific, LLC., USA). Such flocculated clay suspension was then transferred by a pipette to 10 to 15 clear and transparent Petri dishes to allow settling of particles for at least 24 h. Then each Petri dish was placed in a FemoTools FT-UMS 1002 universal measurement stand with a digital microscope (Nanoscience Instruments, Inc., Pheonix, AZ, USA) to capture > 20 images on different locations at the Petri dish bottom surface for broader but no overlapping coverage, to ensure better accuracy and representativeness of the imaged particles.

Image analysis using Image J, an open-source, Java-based software developed and distributed by the U.S. National Institutes of Health (NIH), was conducted to measure the particle sizes, using the widely-accepted systematic procedures^62–64^. It basically involved a few key steps: (1) converting the original color images to 8-bit grayscale counterparts; (2) subtracting the uneven background light; (3) contrasting and segmenting to differentiate the particle boundary; (4) thresholding for producing a binary image of black colored particles with a white background; (5) measuring particle sizes by the built-in “Analyze Particles” function. These steps were herein adopted as the reference procedures for analyzing the captured images of illite flocs settled to the bottom of each Petri dish. The equivalent circular area diameter, *d*_s_, was selected to represent individual particle or floc sizes, which can be calculated by:

12 $$d_{S} = \sqrt {\frac{4S}{\pi }}$$

where *S* is the area of the particle measured by image analysis. For each of the pH values, particle size data collected from all > 20 captured images were merged into a unified dataset, resulting in three separate datasets containing 10,411, 5,357, and 3,285 data entries for the pH 8.61, 4.51, and 2.25 suspensions, respectively, which were then analyzed by the BSI method. Finally, the *K*_max_ selected for these three datasets were 11, 9, and 9 for the pH 8.61, 4.51, and 2.25 illite suspensions, respectively.

## Results and discussion

### Analyses of synthetic datasets

**Figure 1:**
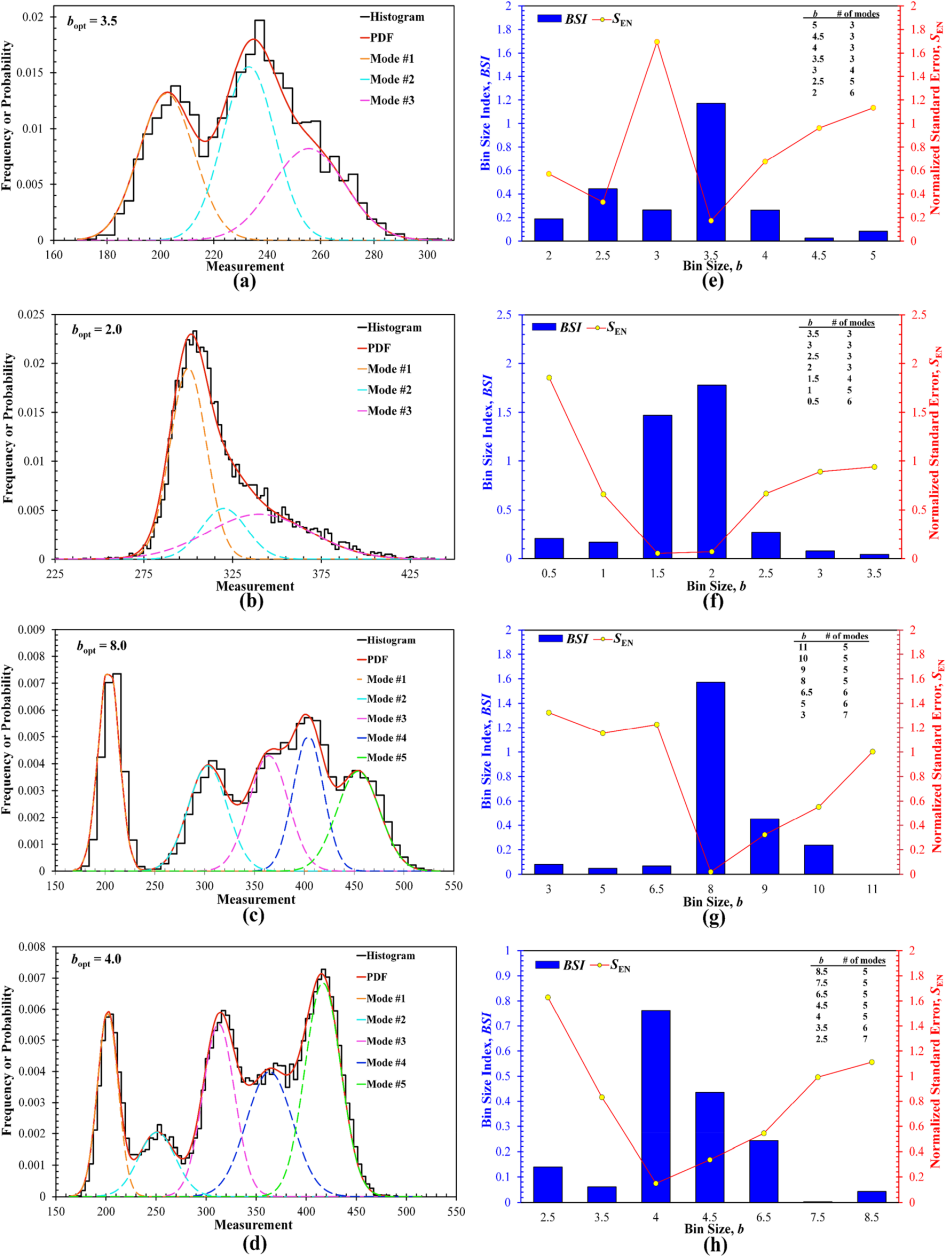
Deconvolution results for the four synthetic datasets: (**a–d**) histogram constructed at the *b*_opt_ and pertinent deconvoluted PDF distribution for Dataset I, II, III, and IV, respectively; (**e–h**) the relationships between the BSI and bin size and between the normalized standard error *S*_EN_ and bin size for Dataset I, II, III, and IV, respectively; The global BSI maximum is used to select the *b*_opt_. Inset tables summarize the number of modes determined by deconvolution for each trial bin size.

The BSI method-derived deconvolution results of the four synthetic datasets (i.e., I, II, III, and IV), consisting of the number, mean, SD, and fraction of all modes, are compared in Table 1 with the known counterparts used for generating the random numbers making up each respective dataset, while Fig. 1 shows the histograms and deconvoluted PDF plots for the *b*_opt_ and the relationships of the BSI versus *b* and *S*_EN_ versus *b* for all four datasets. Also, for each trial bin size *b*, the pertinent deconvoluted number of modes *K* is also summarized in Fig. 1. The initial bin size *b*_0_ determined by Eq. (4) for the four synthetic datasets are 5.0, 3.0, 11.0, and 8.5 respectively, while the corresponding *b*_opt_ are 3.5, 2.0, 8.0, and 4.0, respectively (Fig. 1).

According to Table 1 and Fig. 1, the deconvoluted number of modes (i.e., *K* = 3 for Datasets I and II, and *K* = 5 for Datasets III and IV), and the mean, SD, and fraction of each mode agree remarkably well with the pre-set counterparts, validating the BSI method’s accuracy and effectiveness. First, the fitting PDFs and histograms are consistent, and the number of modes deconvoluted at the *b*_opt_ is exactly the same as the pre-set initial *K*, especially for Datasets II and III that contain densely overlapped modes (Fig. 1b, c). Second, based on the BSI versus *b* plots (Fig. 1e–h), multiple local but smaller BSI peaks exist in all figures, which seems not to affect the selection of the *b*_opt_ corresponding to the global maximum.

**Figure 2:**
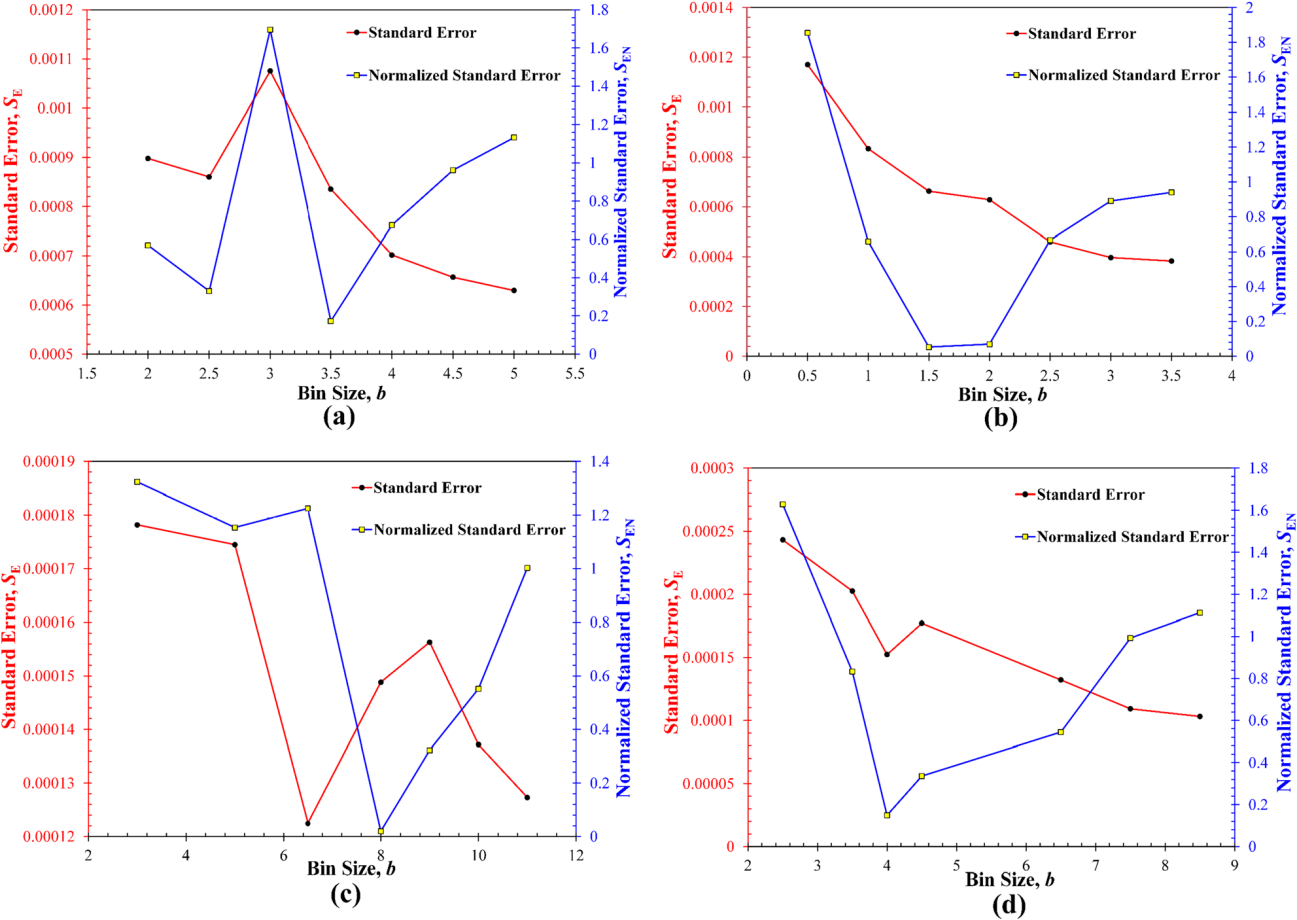
Comparison of the dependence of the standard error *S*_E_ and normalized standard error *S*_EN_ on the trial bin size for the four synthetic datasets. (**a**) Dataset I; (**b**) Dataset II, (**c**) Dataset III, (**d**) Dataset IV.

Although prior work^20^ claimed that the BSI versus *b* plots should exhibit a unimodal peak, it is not uncommon to observe multiple localized peaks in such plots. This discrepancy can be primarily attributed to the variations in the standard error, *S*_E_, obtained from each deconvolution fitting, as shown in Fig. 2 that compares the *S*_E_ and corresponding *S*_EN_ for different *b* values. Clearly, for all four synthetic datasets, the *S*_E_ generally decreases with increasing the *b*, because a larger bin size leads to underfitting (or oversmoothing) of the datasets and hence the reduction of some data features. There still exist different degrees of fluctuations of *S*_E_ as the *b* increases. Also, the penalization to the normalized standard error *S*_EN_ by the number of modes *K* affects the variations of resulting BSI. For example, in Fig. 1, while all BSI show the opposite trend against the corresponding *S*_EN_ (i.e., the highest BSI basically corresponds to the smallest *S*_EN_), Fig. 1f shows that the highest BSI is reached at *b* = 2.0 instead of *b* = 1.5 that yields the smallest *S*_EN_, which is due to the different number of modes at these two trial bin sizes (i.e., *K* = 3 at *b* = 2, while *K* = 4 at *b* = 1.5).

Finally, for all synthetic datasets, the *b*_opt_ selected by the BSI method is usually smaller than the *b*_0_ estimated by the Freedman–Diaconis rule, but close to the median of the several trial bin sizes (Fig. 1), which indirectly validates the core concept of the BSI method: the *b*_opt_ optimized for the construction of appropriate histograms should reflect a rational and successful trade-off between overfitting and underfitting. In fact, the fitting results for those bin sizes > *b*_opt_ tend to underfit the measurement data and hence less accurate due to the smaller number of identified modes, while those bin sizes < *b*_opt_ can also lead to higher fitting errors, as shown by both the *S*_E_ and *S*_EN_ (Fig. 2), which is due to overfitting and the loss of generalization but too many uncaptured local features.

**Figure 3:**
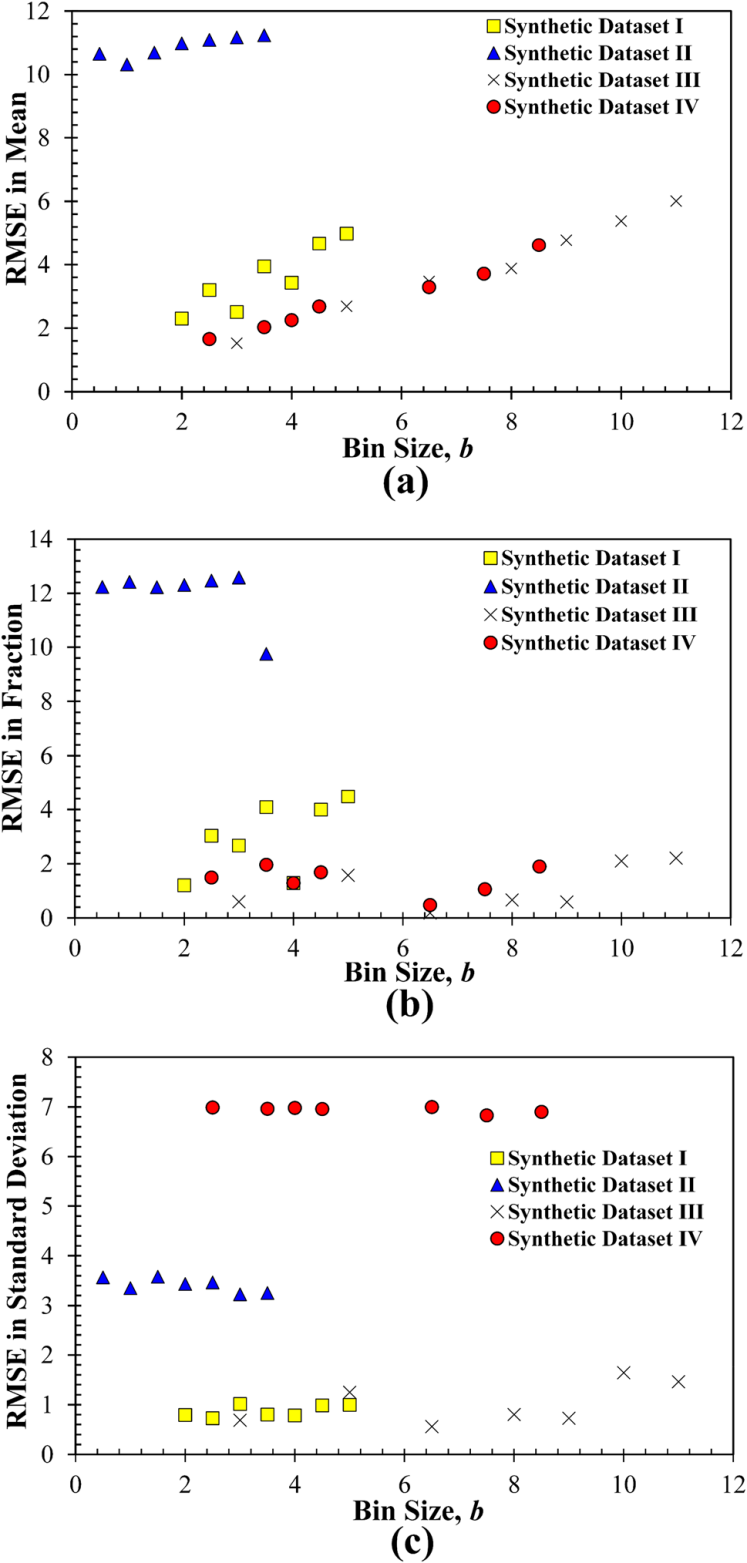
The dependence of fitting errors on the bin size if the number of modes is known and fixed for the four synthetic datasets: (**a**) RMSE in mean; (**b**) RMSE in fraction; (**c**) RMSE in standard deviation; RMSE is the RMS of errors between the deconvoluted PDF and measurement histogram.

It is also noteworthy to discuss how the *b* affects the fitting errors for each mode, including its mean, SD, and fraction. In this regard, since the number of modes in each synthetic dataset was known, the fitting errors were determined based on a fixed mode number (i.e., 3 or 5 for the four datasets) but varying the bin size. That is, the effects of *b* on the accuracy of estimating the modes’ statistical parameters (i.e., mean, SD, and fraction) under a constant *K* were examined. The root-mean-square (RMS) of errors (RMSE) between the given and deconvoluted means, SDs, and fractions (Table 1) of all modes for each trial *b* was calculated, and results are compared in Fig. 3. The RMSE in the mean values of each dataset increases nearly linearly or is generally dependent on the bin size. This phenomenon agrees well with the common understanding that larger bin sizes tend to underfit the data, but help reduce the noises resulting from the measurement randomness at the expense that the deconvolution accuracy for the parameters is lowered, while smaller bin sizes usually yield higher precision (or even overfitting). However, such a trend is not observed for the RMSE in the SDs and fractions for all four datasets. That is, the RMSE of the SDs or fractions is relatively independent on the bin size. Therefore, for a given dataset with the known mode number *K*, if PDF deconvolution is conducted, the deconvoluted SD and fraction of each mode are less affected by the bin size, but an appropriate bin size is required to estimate the more accurate mean of all modes.

In summary, the *b*_opt_ selected by the BSI method yields rational histograms that lead to reasonable and accurate deconvolution results, including the number of modes and three statistical parameters of each mode. The BSI at a given bin size is primarily dependent upon the corresponding *S*_E_, further adjusted by the normalization effects of all *S*_E_ values (i.e., *S*_EN_) and penalized by the number of modes *K*. Thus, when a constant *K* is used for all trial bin sizes, the penalization effects of *K* on the deconvolution results are theoretically negligible. In most data analyses and applications, however, the *K* is usually unknown. As such, a maximum *K* is required to make sure that the *DOF* is greater than or equal to 1. As discussed later, other accompanying measurements (such as XRD discussed in the next section) can help select a *K*_max_. Therefore, the total number of fitting cases is limited for a given trial bin size, which simplifies the entire deconvolution process. As a result, a significant advantage of the BSI method is that it yields both the optimal bin size *b*_opt_ and number of modes *K*, as well as other routine parameters.

### Analyses of datasets on rocks’ elasticity

**Figure 4:**
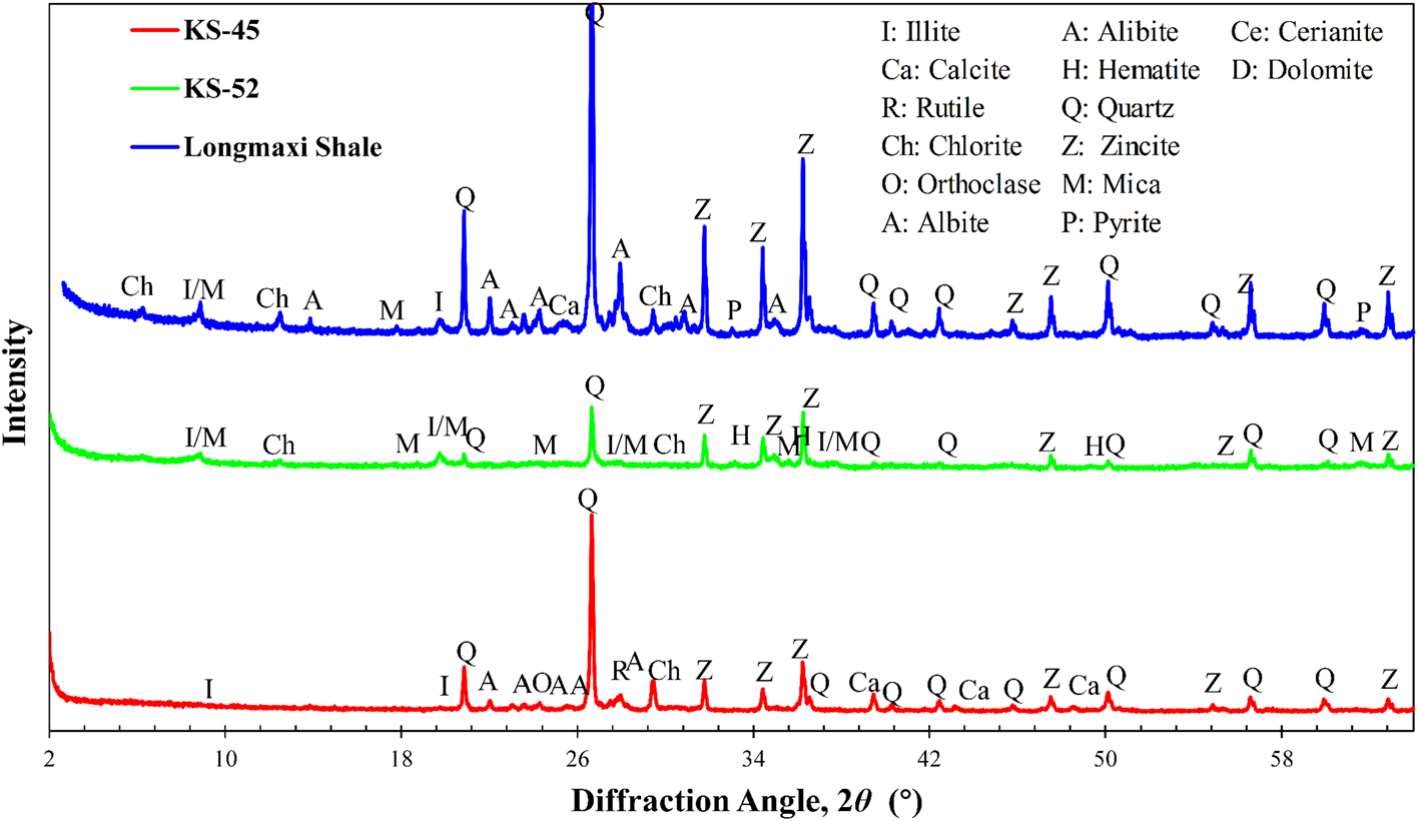
XRD patterns of the three studied rock samples.

Figure 4 presents the obtained XRD patterns of the two sandstones and one shale with all reflections labeled for the identified mineral phases, and quantitative weight-based fractions (wt.%) are accordingly summarized in Table 2. With the known specific gravity *G*_s_ of different minerals reported in the literature^65,66^, the volume-based fractions (vol.%) can then be determined. These results, including the number of modes (i.e., phases) *K* and fractions *A*_j_ can be in turn used as the initial trial inputs for the PDF-based deconvolution of the elasticity datasets of these rocks obtained by nanoindentation.

**Table 2:** Results of qualitative and quantitative mineralogical analyses by XRD for the KS-45, KS-52, and Longmaxi shale.

Mineral	Ideal chemical formula	Specific gravity	Weight fraction (wt.%)			Volume fraction (vol.%)		
			KS-45	KS-52	Shale	KS-45	KS-52	Shale
Quartz	SiO_2_	2.65	53.23	17.99	43.40	53.50	19.57	43.80
Orthoclase	KAlSi_3_O_8_	2.61	6.06	–	–	6.18	–	–
Albite	NaAlSi_3_O_8_	2.61	18.17	2.31	23.40	18.54	2.55	23.60
Biotite	K(Mg, Fe)_3_(AlSi_3_O_10_)(F,OH)	3.05	–	5.18	–	–	4.90	–
Muscovite	KAl_2_(AlSi_3_O_10_)(F,OH)_2_	2.88	–	25.56	12.00	–	25.58	10.90
Calcite	CaCO_3_	2.71	13.09	1.67	2.30	12.86	1.78	2.20
Illite	KAl_2_(AlSi_3_)O_10_(OH)_2_	2.69	8.10	24.66	10.00	8.01	26.42	8.90
Chlorite	Mg_5_Al(AlSi_3_)O_10_(OH)_8_	3.20	–	19.30	5.20	–	17.38	5.30
Hematite	Fe_2_O_3_	5.26	–	3.33	–	–	1.82	–
Rutile	TiO_2_	4.01	1.35	–	–	0.90	–	–
Pyrite	FeS_2_	5.10	–	–	2.00	–	–	1.30
Organic matter	–	1.06			1.70		–	4.00

Prior to the histogram construction, extra effort is still needed to categorize and combine certain different mineral phases, because of two reasons: (1) nanoindentation with finite but not infinitesimal depths has a constrained resolution of detection limits, and hence very small-sized constituents such as clay minerals (e.g., < 2 and ~ 0.2 μm in planar dimension and thickness respectively), organic matter, and the finer interparticle cementation (e.g., carbonates) or pores, cannot be detected or discerned by nanoindentation^20^; (2) some constituents may exhibit similar mechanical properties (e.g., Young’s modulus), although their crystal structures are totally different and clearly distinguished by XRD as two different phases. As such, a homogenized composite phase, consisting primarily of typical clay minerals (e.g., illite, chlorite) and the similarly-sized pores, pore-filling organic matter, and interparticle cementation, is assigned as a “clay matrix” phase. In addition, some other trace minerals, such as rutile, hematite, pyrite, and calcites, which are present at very small fractions (i.e., KS-52 and the shale have only 1.78 and 2.3 vol.% calcites, respectively), can also be grouped into the clay matrix. On the other hand, some hard minerals (e.g., quartz, feldspar) may have very similar Young’s modulus, and hence can be categorized as one unified phase (e.g., a composite phase “QF” stands for the combined quartz and feldspar). This extra data processing usually leads to the number of phases that is less than the counterpart identified by XRD. Finally, due to the finite indentation depths, a virtual phase, termed as “interface”, which accounts for the measurements from indents located at the boundary between two mechanically dissimilar phases (e.g., a hard phase versus the clay matrix), should also be considered in the deconvolution^20^. Noteworthy is that the volumetric fraction of the virtual interface phase should not be counted toward the total composition, but discarded when calculating the actual volumetric fractions of the real but not virtual phases.

The above pre-processing of XRD data yields the maximum for the number of modes, *K*_max_, used to simplify the PDF deconvolution, which is 9, 7, and 8 for the KS-45, KS-52, and shale, respectively. Noteworthy is that these *K*_max_ values are intentionally increased to avoid the missing of some potential modes (i.e., a smaller *K*_max_ might not be able to account for all potential modes). Therefore, for a selected trial bin size, the corresponding histogram can be constructed, and then the number of modes *K* can be determined and optimized by two different approaches: if different individual modes can be clearly discerned and identified from the histogram, then the *K* can be manually determined; In contrast, if the modes are not so clearly separated apart but considerably overlapped, then different *K* values ranging from 1 to *K*_max_ are tried in the deconvolution. Finally, for each combination of *K* and *b*, many sets of initial input parameters, including the mean, SD, and fraction of each mode, are randomly selected and used for each deconvolution fitting, and the final solution is determined by maximizing the BSI.

**Figure 5:**
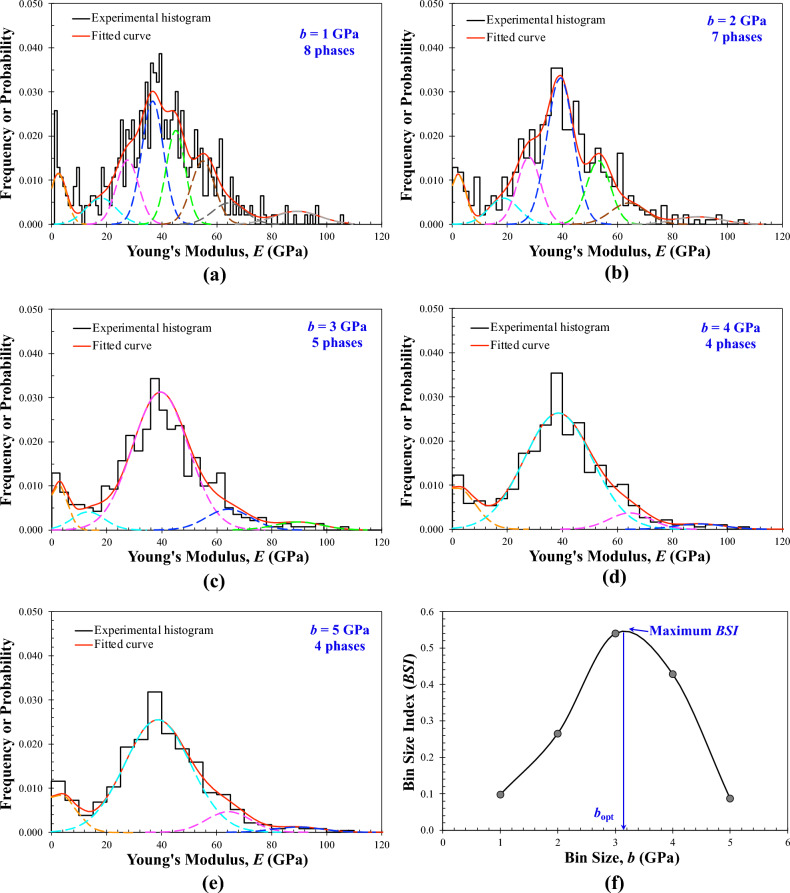
Illustration of the BSI method using the nanoindentation dataset from the shale sample at an indentation depth of 500 nm: (**a–e**) different trial bin sizes of 1.0, 2.0, 3.0, 4.0, and 5.0 GPa and the resulting *K* modes; (**f**) the summary plot of the *BSI* versus trial bin size *b*.

First of all, Fig. 5, using the elastic modulus of the shale rock as an example, illustrates the process of computing and curve fitting that leads to the determination of the optimal bin size *b*_opt_ and hence the corresponding definite mode number of *K.* In Fig. 5a–e, five different trial bin sizes (i.e., *b* = 1.0, 2.0, 3.0, 4.0, and 5.0 GPa) were used to construct different experimental histograms based on the same experimental dataset. Clearly, the shapes of these histograms vary with the bin size. Each of these histograms was then fitted with a multimodal normal distribution function (i.e., the fitted continuous curve), yielding both the errors of fitting and the *K* value (as shown in Fig. 5). Using Eqs. (6) to (10), the BSI corresponding to each trial bin size was computed and then plotted against the bin size, resulting in a unimodal curve (Fig. 5f) of *BSI* versus *b*. The *b* value corresponding to the peak of this curve can then be defined as the *b*_opt_.

**Figure 6:**
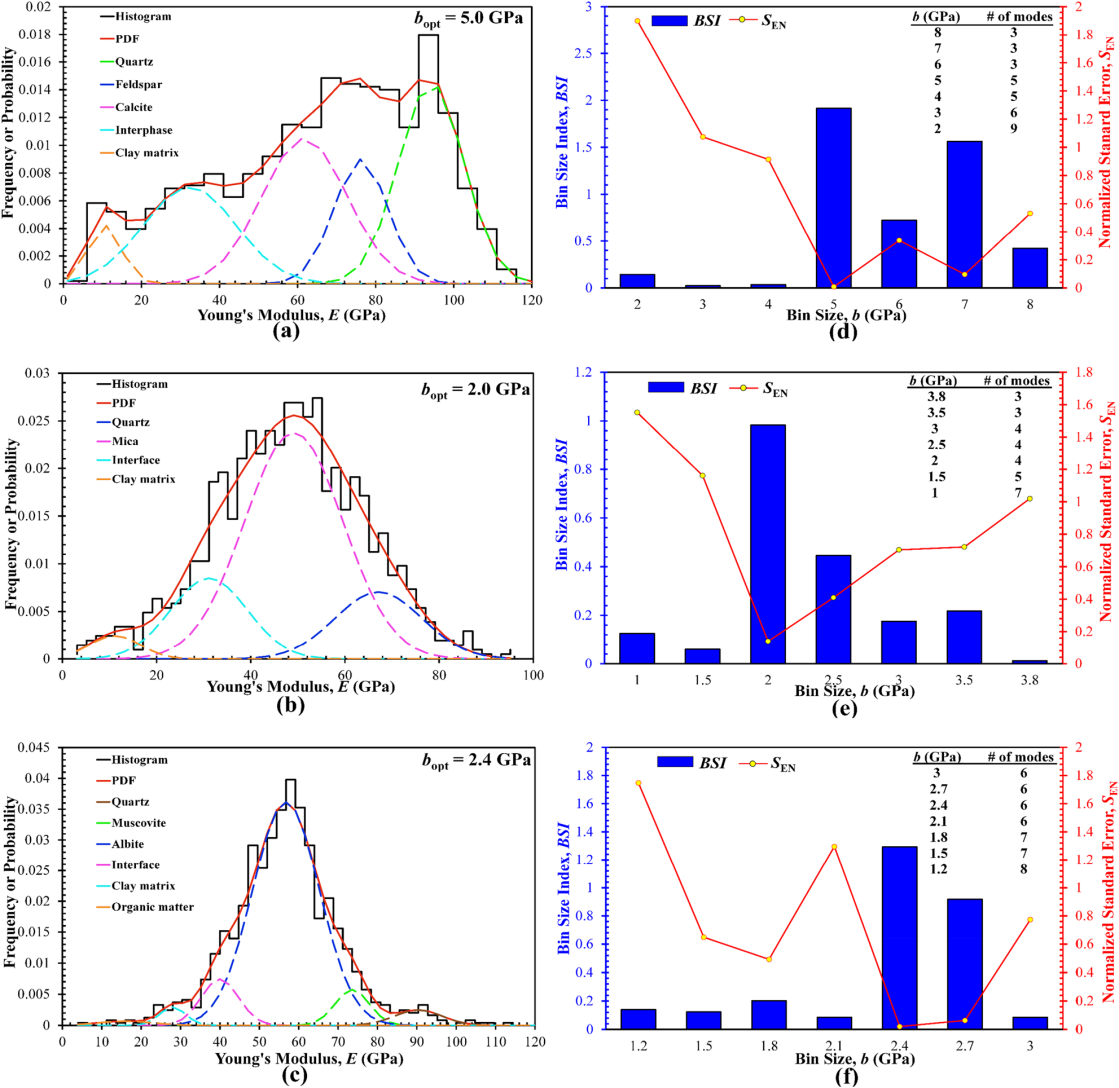
Deconvolution results for the Young’s modulus of three rock samples obtained by statistical nanoindentation: (**a–c**) histogram constructed at the *b*_opt_ and pertinent deconvoluted PDF distribution for KS-45, KS-52, and shale, respectively; (**d–f**) the relationships between the BSI and bin size and between the normalized standard error *S*_EN_ and bin size for KS-45, KS-52, and shale, respectively; The global BSI maximum is used to select the *b*_opt_. Inset tables summarize the number of modes determined by deconvolution for each trial bin size.

**Figure 7:**
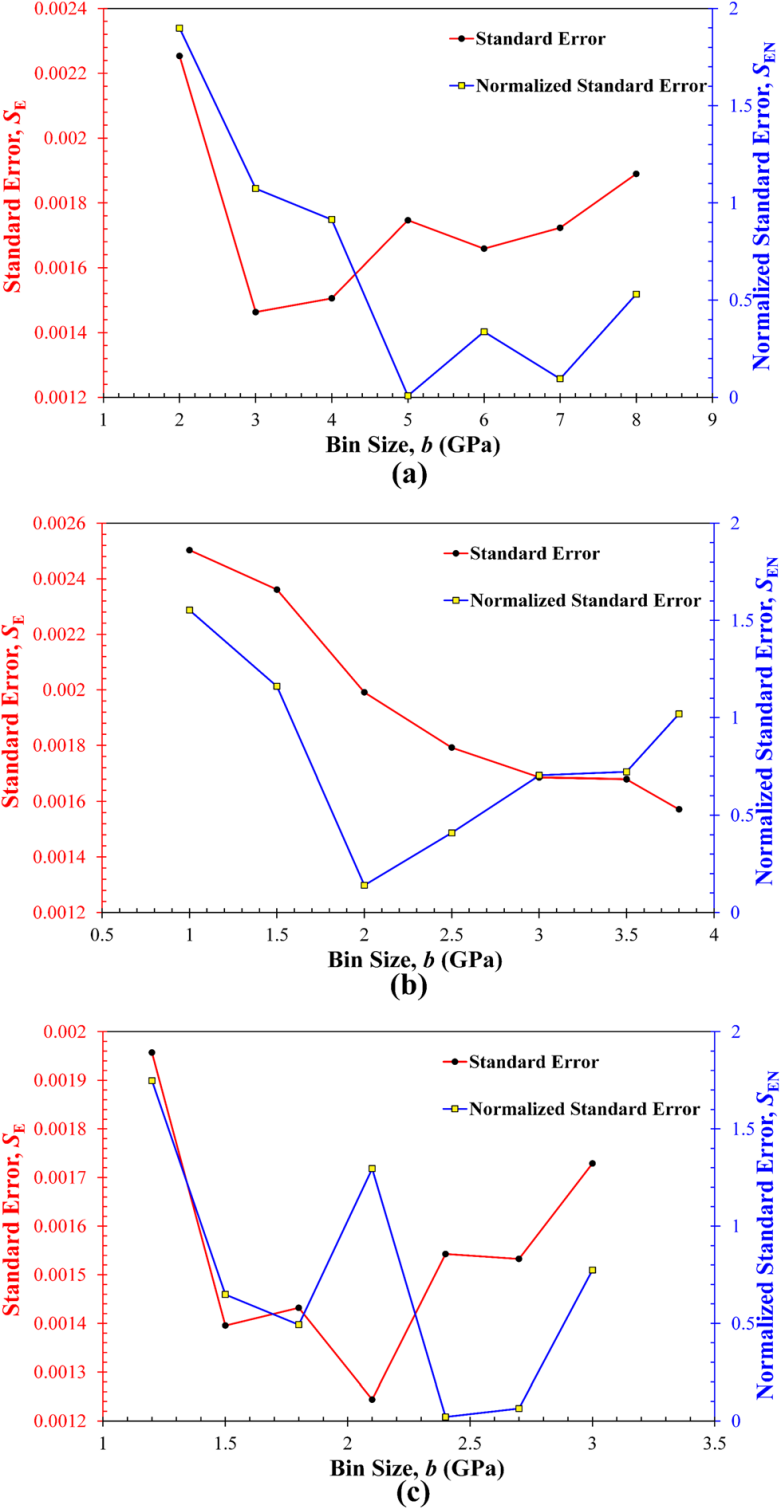
Comparison of the dependence of the standard error *S*_E_ and normalized standard error *S*_EN_ on the trial bin size for the elasticity of three rock samples: (**a**) KS-45, (**b**) KS-53, (**c**) shale.

Figure 6 shows the selected deconvolution results plotted at the *b*_opt_ for each rock sample, together with the BSI versus *b* plot for the selection of *b*_opt_, while Fig. 7 compares the two fitting errors, *S*_E_ and *S*_EN_, obtained at all trial bin sizes. Again, the BSI of each rock exhibits multiple local peaks, as reflected by the varying *S*_E_ and *S*_EN_ in Fig. 7. Nevertheless, while the *S*_E_ of KS-52 increases with decreasing the bin size (Fig. 7b), the other two rocks, the KS-45 and shale, show certain fluctuations in the *S*_E_, although all fittings were performed using the same criterion of maximizing the BSI. This phenomenon may be attributed to the repeated selection of the same number of modes at certain trial bin sizes, particularly the small ones that lead to overfitting and a higher number of modes. Moreover, due to the constraint imposed on each deconvolution fitting to avoid the excessive overlap of any two neighboring modes (Eq. 11), some individual modes that help optimize the global fittings are likely to be omitted, leading to the fluctuations in the resulting *S*_E_. Particularly, for all rock samples, the number of phases or modes determined at their respective *b*_opt_ is consistent with the counterparts determined by the XRD analysis, which thus indirectly verifies the accuracy and effectiveness of the BSI method.

The above deconvolution leads to the identification of distinct modes from the histograms and the mean, SD, and fraction of each mode. By comparing the published elastic modulus of different minerals with the deconvoluted means, each mode can then be assigned to a particular, mechanically distinct phase in the rocks, and hence the in-situ elasticity of different mineral phases is obtained. Table 3 compares the Young’s moduli of all the deconvoluted phases with those reported in the literature^19,20,46,67–71^. Clearly, the Young’s moduli derived from the deconvolution are in excellent accordance with those reported in the literature. Some small discrepancies may be explained by a few factual mechanisms. The property obtained by nanoindentation manifests the in-situ response of the considered mineral phases, which is affected by the residual stress, packing density, and to some lesser extent surrounding phases (i.e., the so-called “indentation surround effect”)^19–21^. For instance, the relatively smaller Young’s modulus of quartz in the KS-52 is likely due to the small fraction (e.g., 19.57 vol.% quartz) as well as smaller sizes of quartz particles, and hence the mechanical response of the quartz to indentation loading is considerably affected by the larger fractions of surrounding finer particles (i.e., the homogenized clay matrix). Such a “surround effect” accounts for the influence of softer phases such as clay matrix on the mechanical response of the hard inclusions surrounded by the former, or vice versa. That is, the in-situ Young’s moduli of the hard inclusions obtained by nanoindentation are reduced by the nearby softer clay matrix that can be included in the expanded elastic zone due to the tiny size of the hard inclusions, even at small indentation depths (e.g., 100–200 nm)^46^.

**Table 3:** Summary of the Young’s moduli (unit: GPa) of individual minerals or phases for the three rock samples determined by statistical deconvolution of nanoindentation data.

Mineral or phase	This study			Reported value (reference)
	KS-45	KS-52	Shale	
Quartz	94.24	67.12	90.39	65.01–105.80^17,41^
Feldspar	76.25	–	–	51.10–85.0^27,42^
Muscovite	–	–	73.59	69.05–77.5^8,19,90^
Calcite	61.79	–	–	64.0 ± 8.00^71^
Albite	–	–	56.79	59.0 ± 3.00^67^
Mica	–	49.12	–	51.0 ± 4.00^46^
Clay matrix	10.62	11.12	27.99	12.0–33.10^91^
Organic matter	–	–	15.99	0–25.00^68^

**Figure 8:**
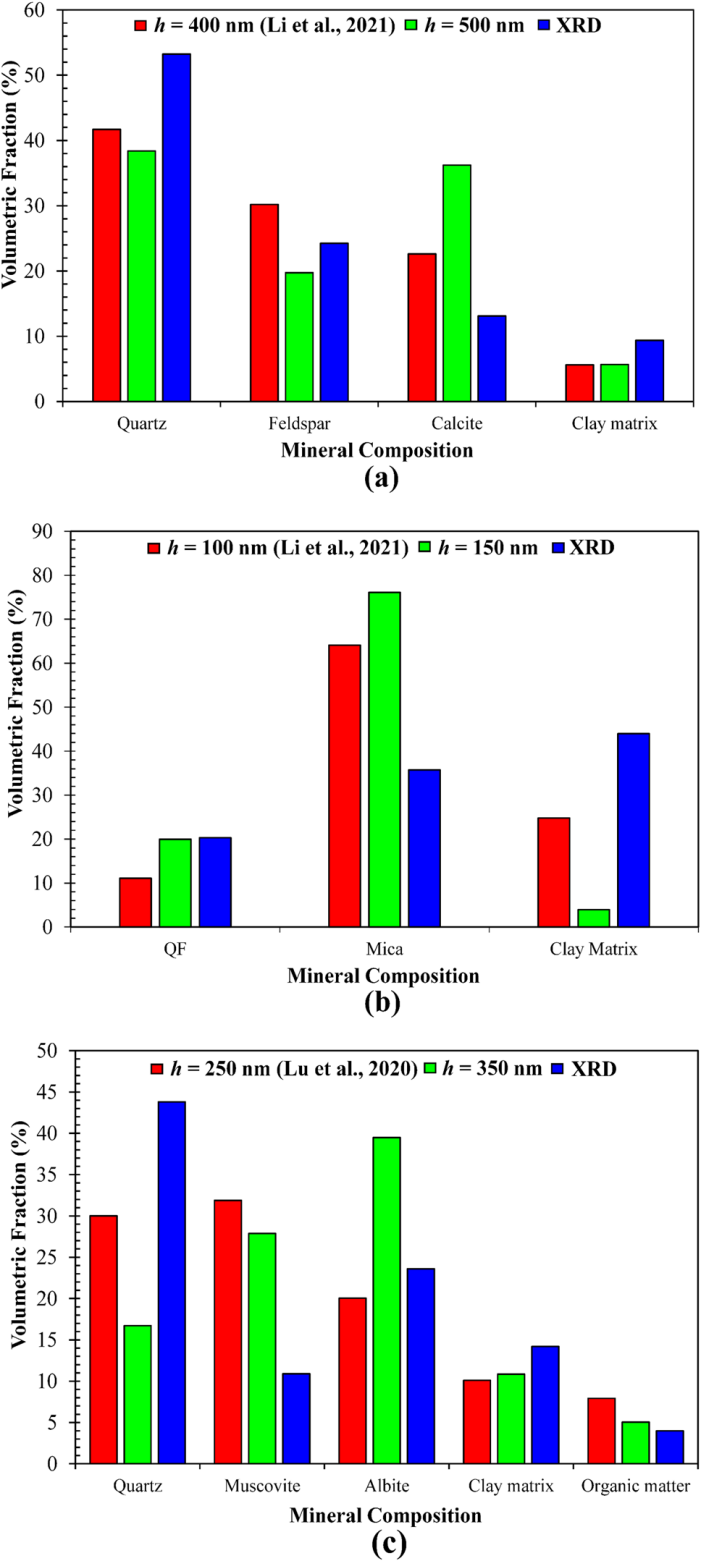
Comparison of the volumetric fractions of different minerals in the three rocks determined by quantitative XRD and PDF-based deconvolution of nanoindentation data at a particular depth *h*. (**a**) KS-45; (**b**) KS-52; (**c**) shale. Reference data at indeptation depth *h* = 400 and 100 nm for the KS-45 and KS-52, respectively, were extracted from Li et al.^17^, and *h* = 250 nm were from Lu et al.^8^.

Another important parameter determined by deconvolution is the volumetric fraction of each mode or phase. Figure 8 compares the volumetric fractions of different modes (or mineral phases) determined by the deconvolution and by the quantitative XRD for all three rocks. In general, the deconvolution results are approximately the same as those from the XRD. The errors and discrepancies may be caused by some tenable reasons. First, XRD is still a semi-quantitative technique. Although it can work well for those inorganic crystalline solid minerals, its quantification of amorphous and/or organic phases (e.g., organic matter or kerogen in oil/gas shales) is usually semi-quantitative and difficult. Second, nanoindentation measurements are size or length-scale dependent, and are incapable of probing the very small-sized crystals and particles such as clay minerals, clay-sized framework silicates (e.g., quartz, feldspar), and oxides (e.g., hematite, goethite). For instance, the fraction of quartz with particle sizes of < 1–2 μm may not be discernable by nanoindentation and hence may be grouped into the clay matrix phase. In contrast, the larger clay particles (e.g., illite and chlorite) with sizes of > 5–10 μm may be detectable by nanoindentation as an independent phase out of the clay matrix. Finally, as pointed earlier, two minerals with distinct crystal structures but similar elastic moduli can be discerned by XRD but not by nanoindentation. Also, due to the indentation size or surround effect, there is a discrepancy between the results from different indentation depths. In fact, the results from two different indentation depths (Fig. 8) are expected to be different. Nevertheless, these two sets of results on the volumetric fractions of various mineral phases are relatively consistent.

### Analyses of datasets on the PSD of flocculated clay suspensions

As stated earlier, PSDs of cohesive suspended matter and natural deposits can generally be described by a lognormal distribution, most often with multiple modes, each of which represents a subordinate lognormal distribution of the sizes of a distinct particle group. To enable the use of the BSI method, routine pre-processing was performed to transform the original lognormal distribution into a conventional normal distribution. That is, if the dataset measured for the variate of particle size *y* follows a lognormal distribution ln[*f*(*y*|*μ*, *σ*)], then the transformed variate *z* = *ln*(*y*) becomes a normal distribution *f*(ln*y*|*μ*, *σ*)^72^. Therefore, logarithmic transformation was first performed on the three PSD datasets collected at different pH, followed by the PDF-based deconvolution with the BSI method. An additional benefit of this logarithmic transformation is to detect the possibly hidden particle size groups due to the high skewness of the lognormal distribution that usually leads to the concentration of data with smaller values.

**Figure 9:**
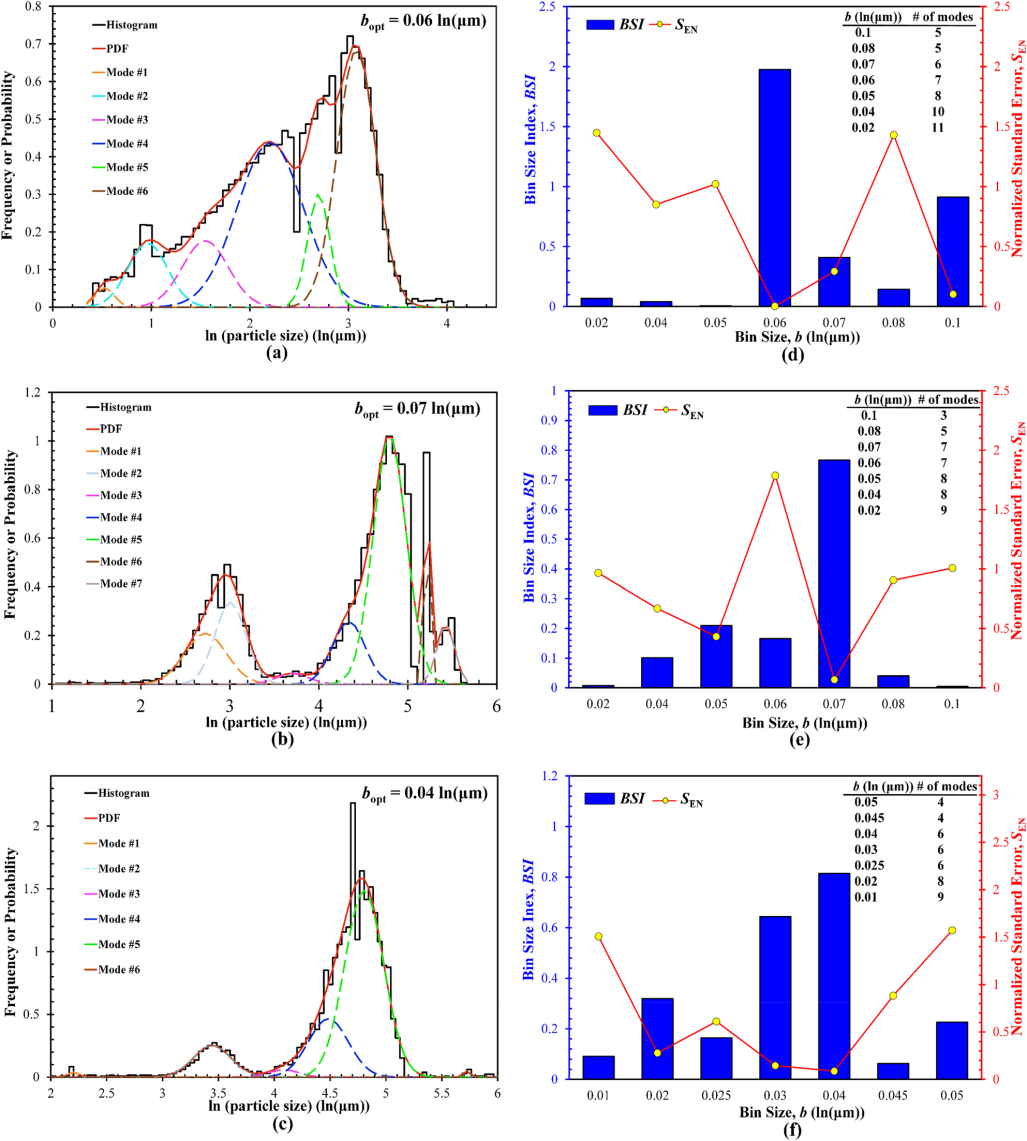
Deconvolution results for the PSDs of three focculated illite suspensions: (**a–c**) histogram constructed at the *b*_opt_ and pertinent deconvoluted PDF distribution for pH  8.61, 5.51, and 2.25 illite suspensions, respectively; (**d–f**) the relationships between the BSI and bin size and between the normalized standard error *S*_EN_ and bin size for pH 8.61, 5.51, and 2.25 illite suspensions, respectively; The global BSI maximum is used to select the *b*_opt_. Inset tables summarize the number of modes determined by deconvolution for each trial bin size.

Figure 9 presents the deconvolution results, including the PSD histograms plotted at the respective *b*_opt_ selected by the BSI method and the comparison between the *BSI* and *S*_EN_. It should be noted here that, unlike the three rock samples that rely on the quantitative XRD analysis to pre-select the *K*_max_, the number of distinct particle size groups (or modes *K*) used in the PDF-based deconvolution of the PSD histograms cannot be determined in advance, but via multiple trials to determine the correct number of modes that guarantees the minimal fitting errors. Interestingly, the total number of deconvoluted distinct-sized particle groups is higher than that reported in some prior studies considering similar materials and environmental conditions (e.g., pure clay minerals and clay-EPS mixture suspensions in saline or fresh water affected by hydrodynamic shearing) as the substitutes for natural cohesive sediments^14,61,73–77^. Such a discrepancy can, to some extent, be due to the logarithm transformation that uncovers the hidden peaks in the originally lognormally-distributed PSDs. Also, the sizes of irregular particles or flocs are defined in this study as the equivalent circular diameter of the particle area measured by optical imaging (Eq. 12). In prior studies, however, different definitions were adopted to represent the measured particle or floc size. For example^14^, calculated the particle or floc size based on the volume moment mean value or De Brouckere mean diameter, as expressed with the following equation:

13 $$d_{V} = \frac{{\sum {pVl} }}{{\sum {pV} }} = \frac{{\sum p l^{4} }}{{\sum {pl^{3} } }}$$

where *V* and *l* are respectively the volume and equivalent sphere diameter of a particle or floc, *p* is the total number of particles or flocs at a fixed size range (i.e., equivalent to the bin size), and *d*_V_ is the mean diameter of all particles or flocs within that given size range. The use of such a mean diameter *d*_V_ generalizes the size range for particles that may fall into different particle size groups (i.e., deconvoluted modes), and hence the resulting histograms become smoother with less localized peaks to be deconvoluted and identified.

**Figure 10:**
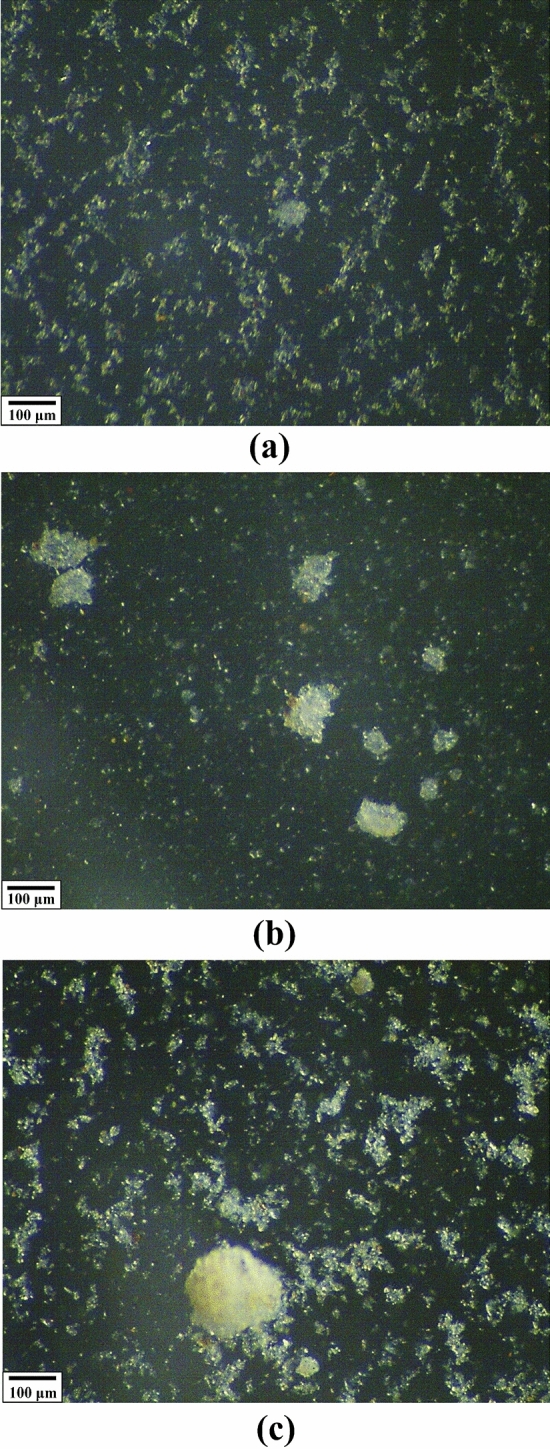
Example optical images of the flocculated illite suspensions prepared at a 35 ppt NaCl salinity: (**a**) pH 8.61; (**b**) pH 4.51; (**c**) pH 2.25.

The means of different-sized particle groups or modes are then collected from the transformed multimodal PDFs shown in Fig. 9, and then further used to calculate the corresponding particle sizes, which manifests a wide, discrete range of particle sizes. Prior studies attempted to conceptually define the particle size groups of suspended cohesive sediments affected by flocculation and hydrodynamic shearing^13,14,58,78^. Since a standard classification of different particle/floc size groups is yet to be developed, the four-level hierarchical particle size system^14^ is followed in this study, and the above deconvoluted modes are assigned to different classified size groups (Table 4). Clearly, the pH of a clay suspension can significantly affect the size kinetics of saline illite suspensions: with decreasing the pH, larger-sized particles or flocs (e.g., macroflocs and microflocs) tend to form, while the population of the finer-sized particles (e.g., primary particles and flocculi) decreases. At pH 2.25, the flocculi group with a size range of ~ 10 to 30 μm even disappears (Fig. 10). In addition, based on the fraction of each size group, the flocculi and primary particles groups can be regarded as the basic constituents of illite suspensions, which make up the entire, original basic illite suspension (i.e., without acid titration to decrease the pH) and interact with each other or even smaller primary particles to form larger microflocs and macroflocs. Similar findings were also reported in the literature, which reveals the mechanisms of flocculation or stability of aqueous colloidal systems affected by the surface properties and water chemistry (e.g., ionic strength, pH, polymeric electrolyte concentrations)^55,73,79–84^.

**Table 4:** Summary of the fractions of different particle size groups in flocculated illite suspensions determined by the statistical deconvolution (note that the sum of fractions for ass particle size groups is 1.00).

Particle group	Size range (μm)	Population fraction, *A* (%)		
		pH 8.61	pH 4.51	pH 2.25
Primary particles	< 10	57.37	–	0.45
Flocculi	10–30	42.63	26.62	–
Microflocs	30–200	–	13.56	33.66
Macroflocs	> 200	–	59.82	65.89

As a typical weathering product of K- and Al-rich sedimentary rocks (e.g., shales), illites are formed by the alteration of muscovite and feldspar under high pH conditions during which highly complex nanoscale irregularities, high crystal defect, and broken bond can develop on the face and edge surfaces of illites^85^. These structural features render illite a very high net negative layer charge (e.g., typically −1.68 per O_20_(OH)_4_) and alkaline properties when dispersed in water, resulting in strong electrostatic repulsion between the face surfaces of individual particles caused by the repulsive double layer, which is further increased due to the pH-dependent negative charge that can also form electrical double layers on the edge surface^86^, and hence stabilization of the clay suspension is achieved by preventing particles from flocculation or aggregation. Increasing the ionic strength by adding different electrolytes (e.g., 35 ppt NaCl salinity selected in this study) and decreasing the pH by adding HCl can both promote flocculation among illite particles and flocs, leading to the formation of larger-sized microflocs and macroflocs, via reducing the repulsive double layer thickness and reversing the negative edge charges into positive ones when the adjusted pH is smaller than the point of zero charge of the illite, respectively, which can both increase the prevailing attractive forces (e.g., Coulomb attraction) over the double layer repulsions (which is also known as DLVO theory)^87,88^.

## Discussion

The above analyses of the synthetic datasets as well as real-world materials characterization datasets on the elasticity of three rocks and on the PSDs of flocculated clay suspensions validate the accuracy and effectiveness of the newly developed BSI method and its applicability to common data processing practices, especially for those involving multimodal datasets. Prior to further discussion, it is worth summarizing the basic concepts and underlying algorithms of this new statistical data binning criterion. A prior common binning criterion, the Freedman-Diaconis rule^31^, in general sets the upper bound *b*_0_ for the optimal bin size, but fails to provide a comparative feedback to assess the errors of the deconvolution or fitting of the histogram constructed by this *b*_0_. In contrast, the BSI method employs a simple comparative, feedback algorithm to select the *b*_opt_ that basically results in the smallest *S*_EN_, which is further penalized by the number of modes *K* (i.e., the errors are also dependent upon both the *b* and *K*), finally leading to the determination of the maximum BSI. In particular, the standard error *S*_E_ obtained at each trial bin size *b* is not treated independently, but weighted and normalized by the mean *μ*_S_ and SD *σ*_S_ of all *S*_E_ values from all trial bin sizes via the *S*_EN_. Different from previous methods and theories that select a bin size by mainly considering the fitting error resulting from only one selected bin size, the BSI method offers a quantitative estimate that delineates the boundary (i.e., the *b*_opt_) between the overfitting with a too small bin size and the underfitting with a too big bin size (i.e., lower precision to the estimation of true statistical distributions).

In this algorithm, the number of unknown parameters, *k* = 3 *K* – 1, is used to balance or penalize the maximum likelihood value *L̂*. Given this theoretical basis, the number of modes or phases used in the PDF-based deconvolution generally varies with the bin size: while a smaller number of modes is usually needed for a larger bin size, more modes are necessary for a smaller bin size to capture more details of the true distribution, and the random (or same) number of modes tend to be selected depending upon the generated *S*_E_ at a given trial bin size. As such, concerns with the correct number of modes selected to determine the smallest *S*_E_ may arise, since an infinite number of modes can be found at any bin size in the deconvolution constrained by only one limited compatibility condition (i.e., Eq. 2, the sum of the fractions of all individual modes should equal to 1.0)^14,21^. This issue, admittedly, reflects the disadvantages of other conventional deconvolution methods (including both PDF and CDF-based methods), which not only require the number of modes as the input parameter for the fitting, but also an accurate estimation of initial values of the three statistical parameters (i.e., mean, SD, and fraction) of all different possible modes. This is particularly important for the gradient descent method that is commonly used for deconvolution to optimize the objective function. Despite the limitation, the validation based on the analyses of synthetic datasets with a constant number of modes still yields an interesting finding. When the number of modes is known and fixed during the deconvolution, the error in the deconvoluted means shows a stronger dependence on the bin size than the counterparts of the other two parameters, SD and fraction. This phenomenon can at least serve as a feasible rule for the prediction of errors in the mean of each deconvoluted mode when the same number of mode *K* is sometimes selected for different trial bin sizes (e.g., prediction of Young’s modulus in the statistical nanoindentation). That is, for a given multimodal dataset, if the mode K is fixed, the error in the means of all *K* modes increases with the bin size.

Furthermore, it is interesting to compare and benchmark the accuracy and rationale of the BSI method with a widely used counterpart, the square root method, a built-in function of Microsoft Excel (Microsoft Office, USA) for bin size estimation (*b*_Excel_) in most routine work:

14 $$b_{Excel} = \frac{{Max\left( {data} \right) - Min\left( {data} \right)}}{\sqrt n }$$

**Figure 11:**
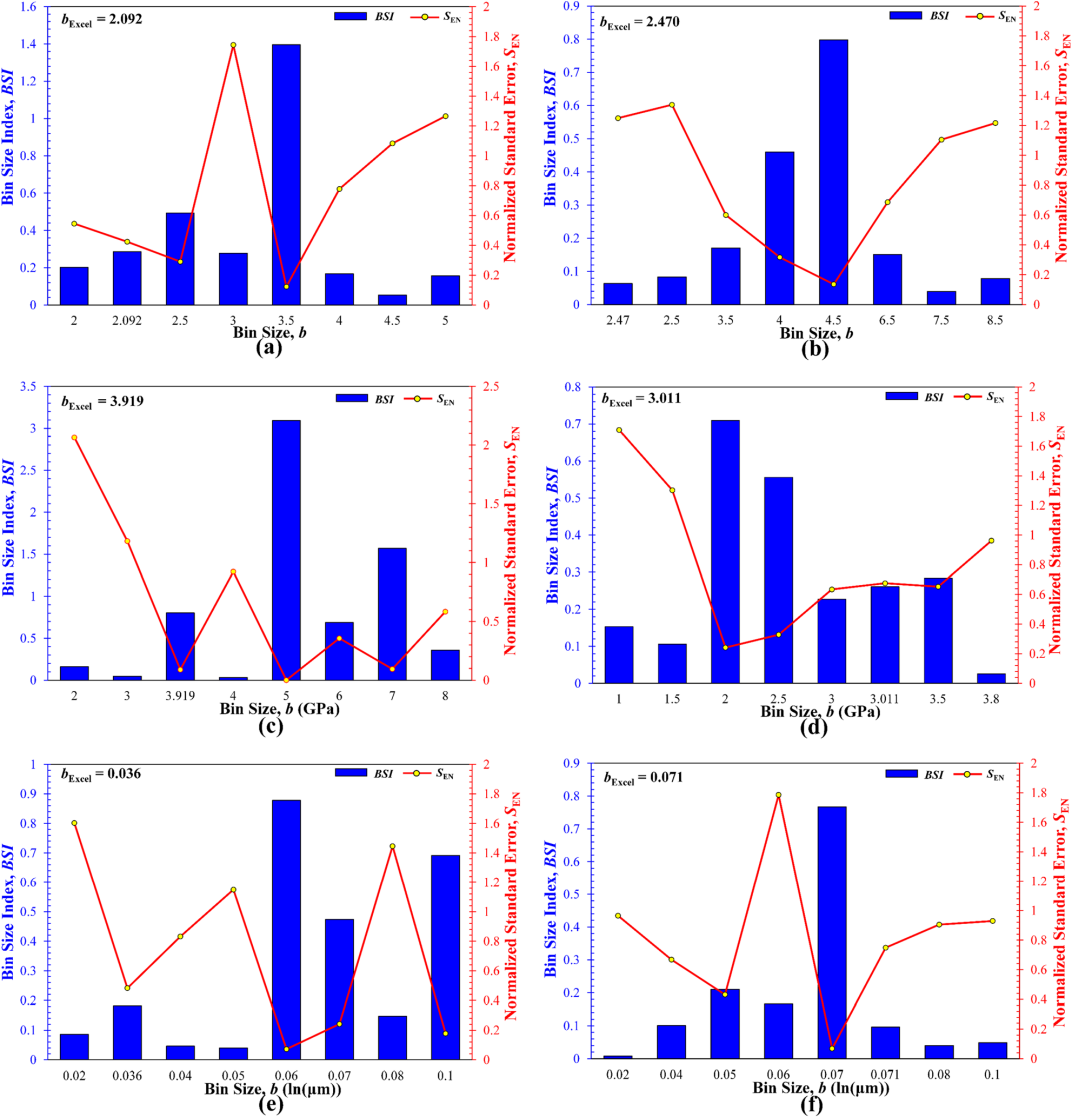
Comparison of the BSI and *S*_EN_ for different bin sizes, including the one determined by the Microsfot Excel program: (**a**) synthetic Dataset I, (**b**) synthetic Dataset IV, (**c**) KS-45, (**d**): KS-52, (**e**) illite suspension at pH 8.61, (**f**) illite suspension at pH 4.51.

This method is used to repeat the deconvolution of some selected datasets discussed above, and results are summarized in Fig. 11. It should be noted that, since the *b*_Excel_ values for the shale and flocculated illite suspension at pH 2.25 are greater than those estimated by the Freedman-Diaconis rule^31^, which serve as the upper bound, deconvoluted results are not compared for these two datasets. For the two synthetic datasets I and IV (Fig. 11a, b), the two *b*_Excel_ values are both much smaller than the *b*_opt_ (i.e., for Dataset I, *b*_opt_ = 3.5, *b*_Excel_ = 2.092; for Dataset IV, *b*_opt_ = 4–4.5, *b*_Excel_ = 2.470), most likely due to the too large *n* values (Table 1). Furthermore, the two *b*_Excel_ values result in the wrong number of modes, i.e., *K* = 6 (versus the correct 3) and 7 (versus the correct 5) for Datasets I and IV, respectively, showing that the *b*_Excel_ cannot find the correct number of modes and hence other three parameters. For the four real measurement datasets (Fig. 11c–f), the *b*_Excel_ is sometimes smaller than *b*_opt_, but could also be greater than *b*_opt_. Nevertheless, the corresponding BSI determined by each respective *b*_Excel_ is not the highest or global maximum, validating that the *b*_Excel_ cannot yield the smallest *S*_EN_ or the highest BSI (see Supplementary Data included in the Excel (Generated Datasets I and IV; Fig. 11) and OriginPro Project (Generated Dataset I.opju and IV.opju) files). Interestingly, in Fig. 11f, a small variation in the *b* (i.e., *b*_opt_ = 0.07 versus *b*_Excel_ = 0.071) can result in a remarkable difference in the BSI, which further indicates that deconvolution should be performed via a trial-and-error algorithm to find the best *b*_opt_, or the selection of *b*_opt_ should not rely on a unidirectional estimation, but more on the feedback of overall normalized errors. In summary, the results from all these *b*_Excel_ values further validate the accuracy and effectiveness of the BSI method in selecting the *b*_opt_ corresponding to the highest BSI for PDF-based statistical deconvolution of multimodal datasets. In addition, as pointed out earlier, the *b*_0_ determined by the Freedman-Diaconis rule is usually greater than the *b*_opt_ determined by the BSI method.

It is worth further discussing the new method’s limitations and advantages. First, the requirements for the datasets such as the number of observations is important for measurements efficiency, duration, and costs. For a *K*-mode normally distributed dataset, there are (3 *K*-1) unknown parameters in the corresponding continuous distribution function (Eq. 1). According to prior work^30,89^, the minimum number of observations or measurements is 20 K*.* However, there is not such a definite maximum number that is then otherwise constrained by other practical concerns such as time and costs of sampling. In general, the larger the datasets, the more accurate the fitting results. Second, only normally or lognormally distributed datasets are analyzed in this study. Although whether this method works for non-normally distributed datasets is unknown, it is postulated that this BSI method is applicable to other statistical distributions that can be approximated or transformed to normal distributions. Future studies should explore similar or equivalent methods for those unconventional statistical distributions.

The novelty of this method lies in two aspects. The first one is the use of the number of modes to penalize the normalized standard error (Eq. 10). For instance, in a typical PDF histogram, a few narrower outlier peaks can be always overfitted with independent modes to minimize the overall standard error, yet they may be measurement errors. Therefore, incorporating the number of modes in the BSI as a penalty to prevent such overfitting can potentially eliminate such tendency. The second one is the calculation of the normalized standard error (*S*_EN_, Eq. 9) which are referenced to all errors from all trial bin sizes, but not one trial fitting only. As such, the value of an individual standard error *S*_E_, which could be very small (although it is difficult to define a criterion for the acceptable values or how small it is small enough), is not too important. Instead, what matters the most is the relative comparison of all *S*_EN_ values. It was found in preliminary trials that a histogram-fitted PDF function with the smallest absolute value of *S*_E_ made little physical sense. For instance, overfitting a histogram with as many modes as necessary can usually lead to a very small *S*_E_ for this individual fitting. However, when all fitting errors are considered as a random dataset with a mean and an SD, its weighting role becomes less important for the entire error dataset. In summary, this BSI method eliminates the criterion for the values of acceptable *S*_E_ and *S*_EN_.

Finally, as binning is widely used to smooth data, handle noisy data, or even more generally to perform data mining (i.e., used as a data pre-processing method to minimize the effects of minor observation errors), the newly proposed BSI method may not only find ample applications to statistical deconvolution of multimodal datasets, but also be expected to play an important role in more generalized data mining and processing practices. For multimodal variates or datasets, an improperly selected bin size usually leads to a wrong number of modes, and hence the deconvoluted results may be misleading. This paper provides a new facile data binning methods and offers an additional alternative to the existing array of processing statistical datasets, particularly those with multiple hidden modes. The extension of the BSI method to two or three-dimensional multivariate datasets warrants further effort, so does its applicability to datasets other than the normal and lognormal distributions.

## Conclusions

This paper presents a new bin size index (BSI) method, developed based on the residual normalized standard error penalized by the number of modes, for binning multimodal datasets for statistical analysis. A total of ten datasets, consisting of four normally-distributed synthetic ones, three normally-distributed ones on the elasticity of three rocks obtained by statistical nanoindentation, and three lognormally-distributed ones on the particle size distributions (PSD) of flocculated illite suspensions, were used to illustrate the BSI method’s concepts and algorithms and demonstrate its accuracy and effectiveness. Based on the above analyses and discussion, the main conclusions can be drawn as follows:

- The accuracy and effectiveness of the BSI method were validated by the synthetic datasets with the pre-assigned number, mean, SD, and fraction of all modes, while the applicability to practical materials characterization demonstrated by the real measurement datasets on the elasticity of multiphase sedimentary rocks and PSDs of flocculated illite suspensions;
- In the plot of the BSI against trial bin size, the global maximum BSI corresponds to the optimal bin size, which can then be used to construct the appropriate experimental histogram required for the PDF-based statistical deconvolution of multimodal datasets;
- The BSI method is demonstrated to be powerful and effective in binning the datasets obeying both normal and lognormal distributions, and is expected to be applicable to other types of statistical distributions;
- For all studied example datasets, the maximum BSI basically corresponds to the smallest normalized residual standard error, but with additional penalization by the number of deconvoluted modes;
- When the number of modes is fixed, the deconvoluted means, but not the SDs and fractions, show a more pronounced dependence on the bin size;
- The optimal bin size determined by the BSI method is not significantly affected by the total number of data entries in the dataset, nor by the maximum, minimum, or range of the data values. Instead, a feedback algorithm is used to compare all fitting errors from multiple trial bin sizes, while other conventional binning methods rely heavily on the number of data entries or range of data values and fail to feedback and assess the fitting errors.
- The bin size plays a more dictated role than the number of modes, since the latter is weakly correlated to, but not totally independent on, the former. Other parameters such as the mean, SD, and fraction of each mode can be determined by the trials of different initial values.

## Supplementary Information

Supplementary Information 1.

Supplementary Information 2.

## Data Availability

All data generated or analyzed during this study are included in this published article and its supplementary information files.
